# Characterizing Mutational Heterogeneity in a Glioblastoma Patient with Double Recurrence

**DOI:** 10.1371/journal.pone.0035262

**Published:** 2012-04-20

**Authors:** Gabrielle C. Nickel, Jill Barnholtz-Sloan, Meetha P. Gould, Sarah McMahon, Andrea Cohen, Mark D. Adams, Kishore Guda, Mark Cohen, Andrew E. Sloan, Thomas LaFramboise

**Affiliations:** 1 Department of Genetics, Case Western Reserve University School of Medicine, Cleveland, Ohio, United States of America; 2 Department of General Medical Sciences (Oncology), Case Western Reserve University School of Medicine, Cleveland, Ohio, United States of America; 3 Case Comprehensive Cancer Center, Cleveland, Ohio, United States of America; 4 Department of Pathology, Case Western Reserve University School of Medicine, Cleveland, Ohio, United States of America; 5 Department of Neurological Surgery, Neurological Institute, University Hospital-Case Medical Center, Cleveland, Ohio, United States of America; H.Lee Moffitt Cancer Center & Research Institute, United States of America

## Abstract

Human cancers are driven by the acquisition of somatic mutations. Separating the driving mutations from those that are random consequences of general genomic instability remains a challenge. New sequencing technology makes it possible to detect mutations that are present in only a minority of cells in a heterogeneous tumor population. We sought to leverage the power of ultra-deep sequencing to study various levels of tumor heterogeneity in the serial recurrences of a single glioblastoma multiforme patient. Our goal was to gain insight into the temporal succession of DNA base-level lesions by querying intra- and inter-tumoral cell populations in the same patient over time. We performed targeted “next-generation" sequencing on seven samples from the same patient: two foci within the primary tumor, two foci within an initial recurrence, two foci within a second recurrence, and normal blood. Our study reveals multiple levels of mutational heterogeneity. We found variable frequencies of specific *EGFR*, *PIK3CA*, *PTEN*, and *TP53* base substitutions within individual tumor regions and across distinct regions within the same tumor. In addition, specific mutations emerge and disappear along the temporal spectrum from tumor at the time of diagnosis to second recurrence, demonstrating evolution during tumor progression. Our results shed light on the spatial and temporal complexity of brain tumors. As sequencing costs continue to decline and deep sequencing technology eventually moves into the clinic, this approach may provide guidance for treatment choices as we embark on the path to personalized cancer medicine.

## Introduction

Human cancers are typically categorized into subtypes based on tissue of origin and/or histopathology. As larger numbers of individual tumors are characterized at ever-higher molecular resolution it is clear that there is substantial mutational heterogeneity across patients, even within the same histopathological subtype. There are two additional levels of heterogeneity that are seldom studied: intra-tumor heterogeneity and heterogeneity of the subpopulations evolving over time. A consequence of intra-tumor heterogeneity is that crucial mutations may be present in only a subset of cells. Moreover, treatments may lead to selective expansion or regression of particular subpopulations within the original tumor, particular when it evolves to a recurrence or metastasis. Researchers have begun to take advantage of such intra-patient heterogeneity, inferring the mechanisms and temporal sequence of events in metastasis in pancreatic carcinoma [1], [2] and breast cancer [3], [4].

Gliomas are known for their inter- and intra-patient heterogeneity, and represent a substantial challenge for the surgeon. A persistent problem during surgery is precise resection of a maximum amount of malignant cells while preserving healthy brain tissue. Glioblastoma multiforme (GBM) is the most common primary brain malignancy in adults, accounting for some 13,000 deaths per year in the United States [5]. In addition to surgery, treatment for GBM patients generally consists of radiotherapy and chemotherapy. Unfortunately, these treatments are rarely curative and the vast majority of tumors recur locally within the brain. At present, it is unknown whether the primary reason for this is lingering malignant cells, *de novo* clonal expansions, selective pressures from adjuvant radiation/chemotherapeutic treatment, or some other mechanism [6]–[8]. Although a subset of patients show increased survival with concurrent radiation and chemotherapy, there is an urgent need for better treatments [9].

To help gain understanding that may lead to new therapeutic options, some recent studies have aimed to identify specific mutations, genes, and molecular pathways driving gliomagenesis. Two such large-scale efforts [10], [11] included in their strategies targeted capillary-based sequencing of genes across large numbers of tumor samples. Performing Sanger sequencing at one tumor region per patient can be problematic, however, because of cell-to-cell molecular heterogeneity within each region, as well as region-to-region heterogeneity within the tumor. Indeed, resected tumor samples are frequently extremely admixed, making accurate detection of the tumor-specific mutational signature non-trivial. Expanding the knowledge of the mutational spectrum of matched sets of primaries and recurrences for patients with GBM may shed light on this molecular heterogeneity and the temporal sequence of mutations that arise in response to the selective pressures of radiation and/or chemotherapy.

Technologies for interrogation of the cancer genome have decreased in cost and exploded in throughput over the last decade. Most recently, “next-generation" sequencing has emerged as a crucial tool in this effort [12]. The new sequencing technologies are capable of producing tens of millions of short (75–100 bp) reads in a single experiment with cost currently around $1,000 and decreasing. Critically, each read represents the sequence of single DNA molecule, rather than the combined signal from all cells in the sample, as is the case in Sanger sequencing. As a result, the new technology is able to detect mutations that are present in a small minority of cells [13]. Here we sought to characterize tumor heterogeneity across various tumor regions as well as over time using a single lane of sequence from a widely-used next-generation sequencing platform. We interrogate seven distinct samples from a single GBM patient, targeting genes known to have important roles in glioblastoma [10], [11], [14]–[16]. Leveraging the platform's power to detect low-frequency mutations, our goal was to characterize heterogeneity with regard to both presence and cellular frequency within each tumor region. This approach may serve as a paradigm for the pursuit of personalized medicine in the setting of brain cancer.

## Materials and Methods

### Ethics Statement

The Case Cancer Institutional Review Board approved all activities pertaining to the study. Written informed consent was obtained from the participant.

### Patient samples and DNA extraction

**Figure 1:**
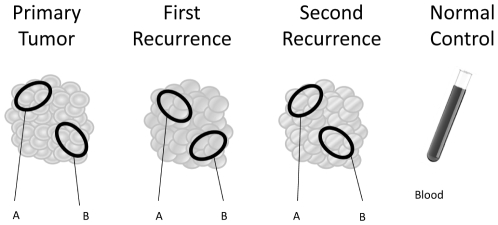
Sample set schematic. This schematic gives a conceptual view of the seven GBM patient samples interrogated in this study. From left to right, we will refer to them in this manuscript as: primary tumor A, primary tumor B, first recurrence A, first recurrence B, second recurrence A, second recurrence B, and blood.

The patient was a male of European ancestry, diagnosed at age 69 with primary GBM and subsequent double GBM recurrence. Treatment for the primary tumor involved surgical resection followed by concurrent radiotherapy and temozolomide chemotherapy, and then adjuvant temozolomide chemotherapy [9]. Treatment for the first recurrence involved surgical resection followed by chemotherapy with thalidomide and bevicizumab. Treatment for the second recurrence involved surgical resection only. Time between primary diagnosis and first recurrence was 6 months. Time between first and second recurrences was 3 months, and overall survival time from primary diagnosis was 11 months. After *en block* resection, pieces of the most representative portion of each tumor were dissected into 0.1–0.2 g aliquots and snap-frozen in liquid nitrogen within five minutes of resection using an IRB-approved protocol. H&E staining was performed by a trained neuro-pathologist using a six-micron re-cut of tumor tissue. Proportion tumor nuclei ranged from 10–60% and proportion necrosis ranged from 10–90%, which is typical in resected brain tumors. This study examined normal blood obtained at the time of diagnosis, and two randomly selected specimens from each of the three resected tumors (Figure 1). DNA was extracted from whole blood and from 25 mg of each snap frozen tumor sample using the QIAamp DNA Mini Kit (Qiagen Ltd) as per manufacturer's instructions for each biospecimen type.

### Gene selection

Since the aim of this study was to characterize mutational heterogeneity, we selected genes that have been reported as being frequently altered in GBM in previous studies. Using published lists of frequently altered genes [10], , we selected ten – *CDKN2A*, *CDKN2B*, *EGFR*, *IDH1*, *IDH2*, *NF1*, *PIK3CA*, *PIK3R1*, *PTEN*, and *TP53* – for exon sequencing.

### Exon amplification and barcoding

Primers were designed using ExonPrimer (http://ihg2.helmholtz-muenchen.de/ihg/ExonPrimer.html) for PCR amplification of exons. Target specificity was assessed using electronic PCR [17]. A total of 107 primer pairs covering 168 exons of 10 the target genes were designed in total (Table S1). Following purification using the QIAquick PCR Purification Kit (Qiagen Ltd; Valencia, CA), PCR products from a single sample were pooled together and sonically fragmented to sizes of 100–200 bp using adaptive focused acoustic technology (Covaris; Woburn, MA). After shearing, adapters including a unique sample-specific barcode 7 bp in length and universal sequencing primers were ligated to either end of the DNA fragment. The resulting ligation products were run on an agarose gel, fragments 150–200 bp were gel-excised and extracted from the agarose, and a second PCR was performed using the universal sequencing primers. Following an additional purifications step, all the bar-coded samples were pooled together and delivered to the High-Throughput Sequencing Core Facility at the Case Western Reserve University Comprehensive Cancer Center.

### Ultra-deep sequencing

Reads of 75 bases were generated using the single-end protocol of the Illumina Genome Analyzer II. Exact duplicates were discarded as potential PCR artifact. The 7-base barcode subsequences were used to uniquely assign each read to its parent sample, then removed *in silico*. The resulting 68-base reads were next base-quality filtered as follows. If all bases had quality scores above 22, the read was left intact. Otherwise, the read was split into 34-base halves. Halves with any base quality score below 23 were discarded. The combined set of 34- and 68-base reads was then aligned to the reference human genome sequence (hg18) using the MAQ software [18], and poor read alignments were removed according to the software defaults.

### Allele frequency and mutation calling

A single-base deviation from the human reference genome sequence within a target exon was flagged as a possible single nucleotide variant (SNV) if, in a sample: (i) it was observed in at least seven independent reads, at least one of which came from each of the positive and negative strands; (ii) at least 10% of the reads carried the same non-reference allele; (iii) the base did not lie within a homopolymer repeat; and (iv) the base did not correspond to an alternate allele in a pseudogene region. The SNV sites were then queried in all other samples. Mutations with allele frequencies below 5% were disregarded as potential sequencing errors.

### Pyrosequencing and TA cloning validation

Pyrosequencing was performed as previously described [19], [20]. Briefly, PCR amplification products from all seven samples were delivered to the pyrosequencing facility in a blinded manner and sequenced using the sequencing primers designed specifically for the PyroMark MD pyrosequencing instrument (Biotage) as per the manufacturer's instructions. Each experiment was performed in triplicate, and the mean of the estimates is reported. For TA cloning, the region harboring the putative mutation was PCR-amplified, and the amplicon was inserted into the pGEM vector (Promega) by ligation with a T4 DNA ligase enzyme. The ligated products were then transformed into DHα5 (Invitrogen) competent bacterial cells. The reaction includes a heat shock step followed by plating onto LB plates with ampicillin, X-gal and IPTG for selection of the transformed product. Positive colonies were used as an innoculum for growing up the clones in 200 µl LB media with ampicillin. To identify the putative mutation in a single clone, sequencing was performed directly from the bacterial cultures on an ABI 3730 DNA Sequencer.

## Results

### Coverage Metrics

After the filtering steps described in the Materials and Methods section, the mean sequence coverage across all samples for the bases in the interrogated exons was 124 reads per base (sample mean range 81–145). Although the sequence coverage varied substantially among genes, coverage within each gene was quite consistent across samples (Figure S1). Differences in gene coverage were therefore most likely either the result of differential PCR efficiency that was consistent across samples, or of varying degrees of non-unique reference sequence within codons.

### Candidate mutations

**Figure 2:**
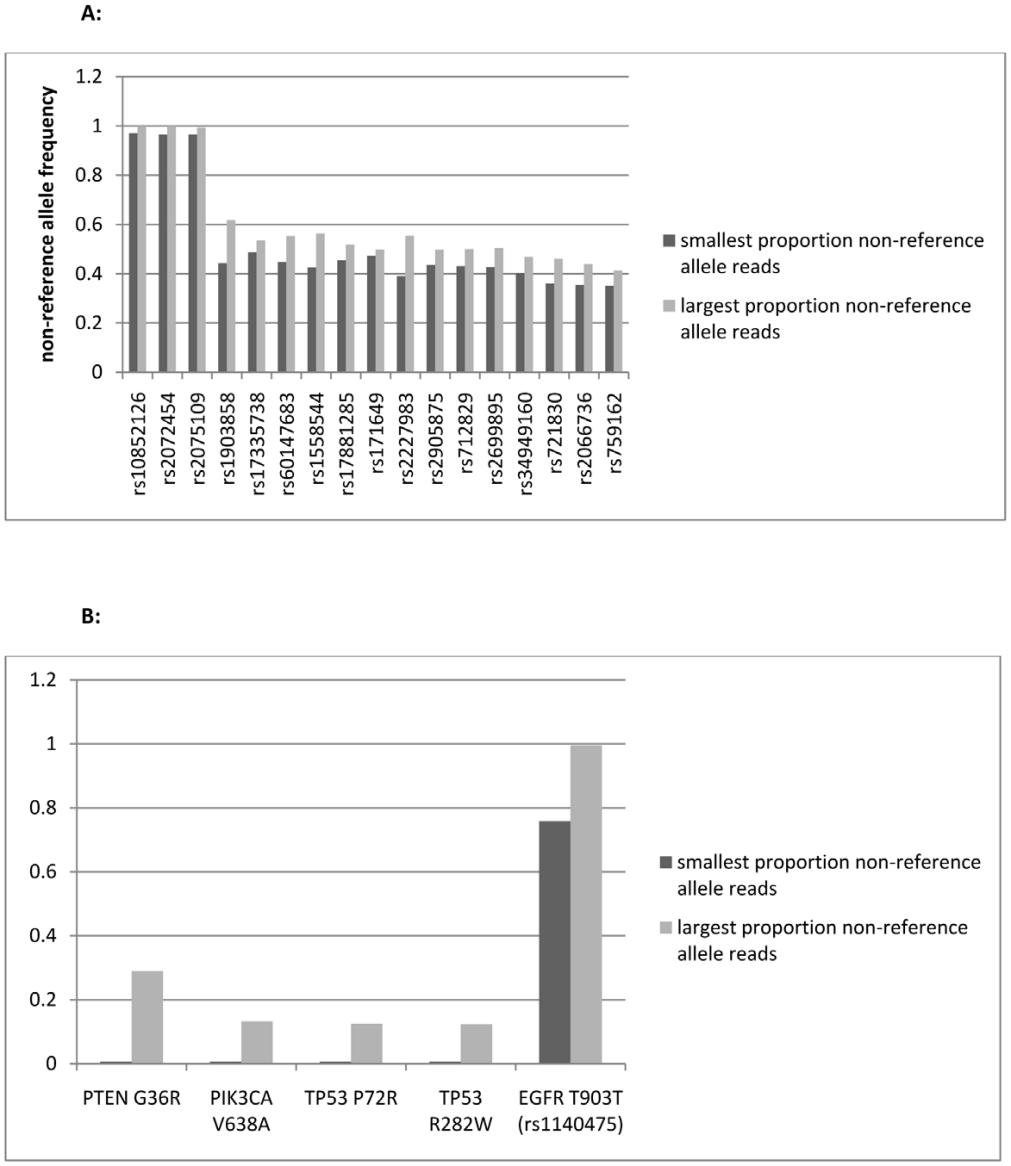
Distinguishing germline variants from somatic variants. (A) For each SNP, the non-reference allele frequencies for samples (out of seven) with smallest (black) and largest (gray) such frequencies are shown. In all samples, these frequencies do not deviate substantially from the germline frequencies. All SNPs in all samples have minor allele frequencies near the expected 50% or 100% and are therefore clearly and consistently distinguishable as either homozygotes (leftmost three) or heterozygotes (the remainder). (B) In contrast, the somatic variants display much wider ranges in allele frequencies across samples. Note that the absence of black bars is indicative complete absence of the mutation in some samples.

Using the criterion described in Materials and Methods, we flagged 22 nucleotide positions as candidate SNVs (Table S2). Of these, 18 were identified in the normal blood (and in the tumor samples) and are all annotated as single nucleotide polymorphisms (SNPs) in the dbSNP database (http://www.ncbi.nlm.nih.gov/projects/SNP/). Such known SNPs provide convenient control sites with which to assess the accuracy of the read allele frequency as a measure of actual sample allelic content. Specifically, in the germline and tumor samples unaffected by copy number aberrations, we would expect non-reference allele read counts near either 50% (for heterozygotes) or 100% (for non-reference allele homozygotes) at these SNPs. This signature is clear throughout all samples in 17 of the 18 SNPs (Figure 2A) which attests to the precision of read count proportion as a measure of true frequency. This left five sites as candidate somatic mutations (Figure 2B and Table 1). These include the remaining SNP, a silent variant in *EGFR* that is clearly a minor allele homozygote in all samples except the second recurrence B, where it reverts to a heterozygote in approximately half of the cells.

**Table 1:** Somatic mutations.

Sample	Gene	Chromosome	Position	Ref	Alt	No. Read	No. Ref	No. Alt	% Alt	Amino Acid Change
Blood	PTEN	10	89643788	G		78	77	0	0	
primary tumor A	PTEN	10	89643788	G	A	122	93	29	23.8	G36R
primary tumor B	PTEN	10	89643788	G	A	52	39	13	25	G36R
first recurrence B	PTEN	10	89643788	G	A	100	71	29	29	G36R
Blood	TP53	17	7517819	G	A	388	383	4	1.0	
second recurrence B	TP53	17	7517819	G	A	405	355	50	12.4	R282W
Blood	TP53	17	7520197	G		163	160	0	0	
second recurrence B	TP53	17	7520197	G	C	215	188	27	12.6	P72R
Blood	PIK3CA	3	180420432	T		25	25	0	0	
primary tumor A	PIK3CA	3	180420432	T	C	23	21	2	8.7	V638A
primary tumor B	PIK3CA	3	180420432	T	C	13	12	1	7.7	V638A
first recurrence A	PIK3CA	3	180420432	T	C	14	13	1	7.1	V638A
second recurrence B	PIK3CA	3	180420432	T	C	53	46	7	13.2	V638A
Blood	EGFR	7	55233911	T	C	669	6	663	99.1	T903T
second recurrence B	EGFR	7	55233911	T	C	603	146	457	75.8	T903T

Read counts in the blood sample are shown as a reference control.

### Independent validation

**Figure 3:**
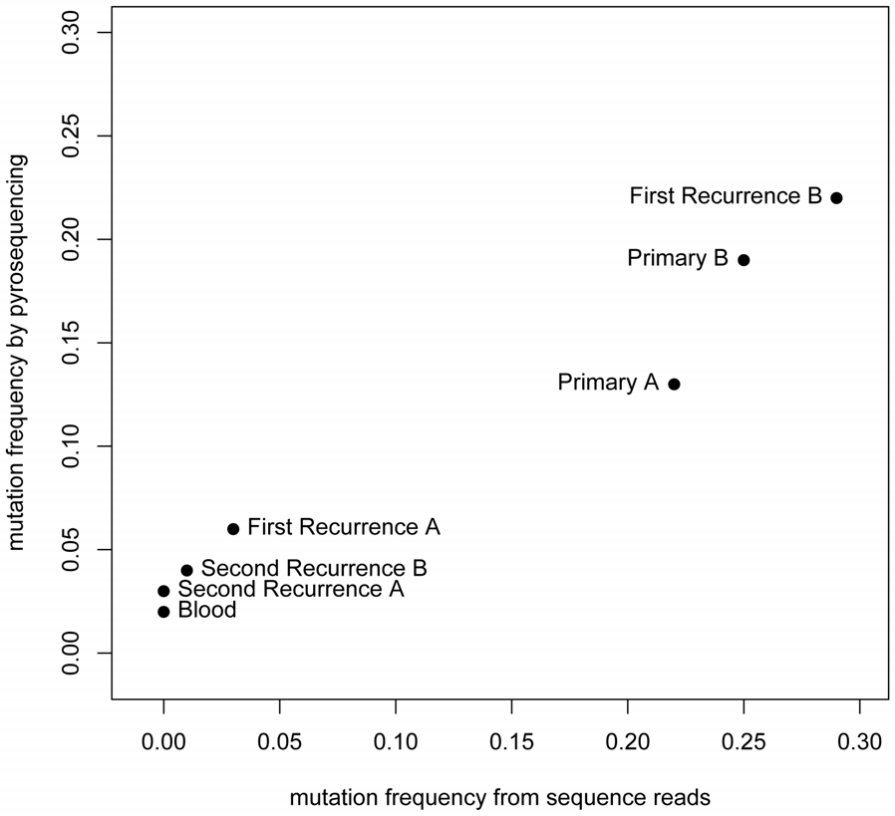
Validation of PTEN mutation. For the sequencing data, the percentage of reads harboring the mutation (horizontal axis) is used as an estimate of the mutation frequency in each sample, plotted against the pyrosequencing frequency estimates (vertical axis; see Materials and Methods).

To guard against false-positive mutation calls that can arise from technological artifacts – particularly in emerging technologies such as next-generation sequencers – we validated somatic alterations using two independent methods. The relatively low mutational frequencies (Table 1) make reliable detection challenging for Sanger sequencing [21]. Seeking to validate both the presence and frequencies of the mutations across all samples, we performed pyrosequencing (see Materials and Methods) of the *PTEN* mutation site in all seven samples. Pyrosequencing allows simultaneous detection and quantification of specific nucleotide residues. For each sample, the resulting mutational frequency estimates were remarkably consistent between the Illumina sequence output and pyrosequencing (Figure 3). We also validated the *PTEN* and *TP53* R282W mutations by sequencing multiple TA clones generated from samples harboring the mutations (Figure S2).

### Prior reports and functional impact of detected mutations

One of the goals in this study was to distinguish between the mutations driving tumor initiation and recurrence from those that are passenger consequences of treatment and/or general genomic instability, or whose presence does not confer resistance to standard therapies. The *PTEN* codon 36 that is altered in three of the samples has been reported by other groups to be quite frequently mutated somatically, particularly in GBM. The COSMIC database [22] cites six cases of GBM with alterations in *PTEN* codon 36, one of which has the same G36R mutation as our study's patient. The Cancer Genome Atlas GBM study [10] reports one sample with a G36E mutation. Regarding *TP53*, the R282W substitution is one of the most common across many tumor types, and is reported in 20 brain tumors in the COSMIC database, as well as in two of the 206 TCGA GBM samples. On the other hand, the P72R mutation is actually at the site of a common SNP, rs1042522 (minor allele frequency 23.3% in HapMap [23] European ancestral samples (CEU)) and has not been previously reported as a somatic mutation. Similarly, the mutated *EGFR* site is a SNP (rs1140475) that has not been previously reported as a somatic mutation, nor has any site in the *PIK3CA* codon 638.

**Figure 4:**
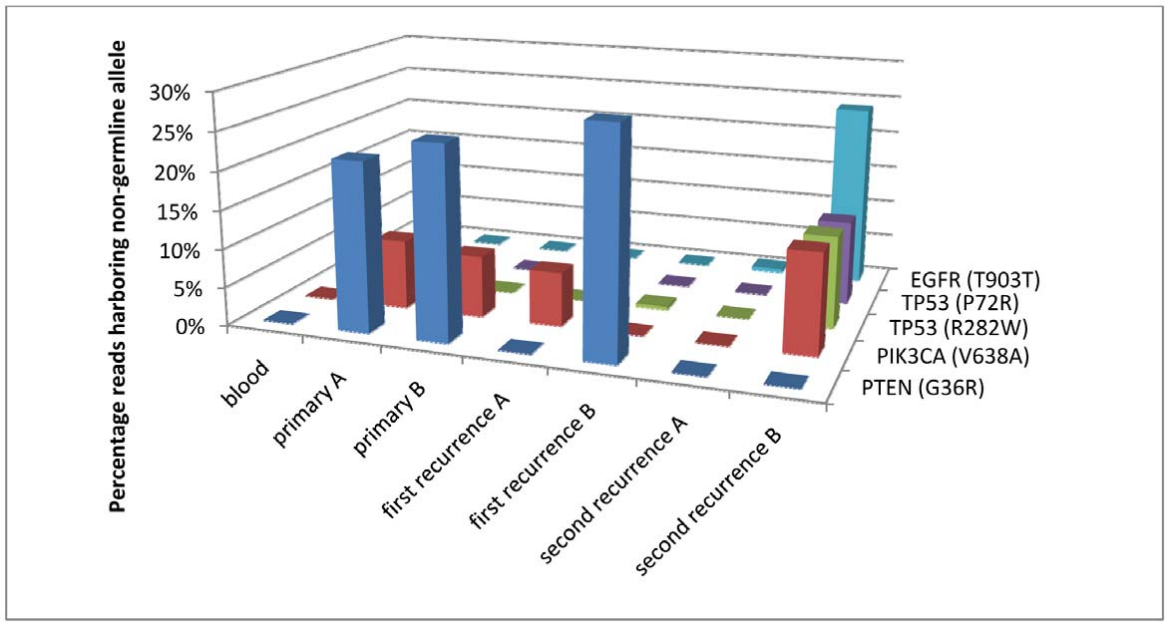
Spatial and temporal mutational heterogeneity. For each sample, the percentage of reads harboring each mutation is plotted. If a mutation is present in a heterozygous constellation, the percentage of cells carrying it will be approximately twice the read percentage displayed here.

To determine whether the somatic substitutions are potentially deleterious, we queried them using three widely-used computational tools: SIFT [24], CanPredict [25], and PolyPhen-2 [26] (Table 2). All of these aim to classify the impact of amino acid changes on protein function by analyzing cross-species conservation of the residue, biochemical properties of the amino acid sequence, curated literature, and other sources. The *PTEN* G36R substitution that dominates the mutational landscape (Figure 4) of the primary and first recurrence samples is classified as deleterious by all four tools. However, as the patient progresses to the second recurrence, three of the four other mutations were either silent or classified as benign, with only the *TP53* R282W substitution deemed deleterious.

**Table 2:** Computational classifications of mutations.

Gene	Chromosome	Position	Amino Acid Change	SIFT Prediction	PolyPhen Prediction	CanPredict Prediction
PTEN	10	89643788	G36R	DAMAGING	Probably Damaging	Likely Cancer
TP53	17	7517819	R282W	DAMAGING	Probably Damaging	Likely Cancer
TP53	17	7520197	P72R	TOLERATED	Benign	Likely Not Cancer
PIK3CA	3	180420432	V638A	TOLERATED	Benign	Likely Not Cancer
EGFR	7	55233911	synonymous	N/A	N/A	N/A

### Contrasting mutational and histopathological landscapes

**Figure 5:**
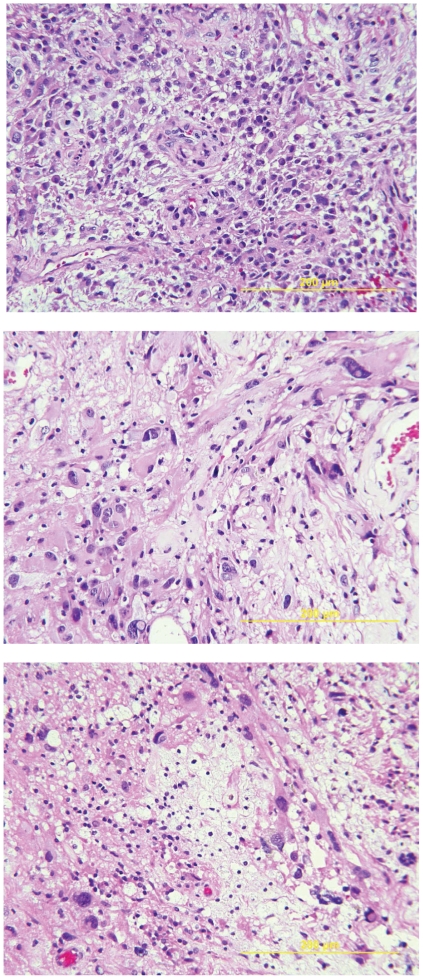
Histopathology of the primary tumor and recurrences. The primary tumor (top) shows uniform cellularity with moderate pleomorphism. The first recurrence (middle) demonstrates marked nuclear pleomorphism and regions of necrosis, while the second recurrence (bottom) shows enlarged, moderately pleomorphic nuclei.

Having established mutational heterogeneity among our set of patient samples, we sought to determine the degree to which the heterogeneity was reflected in the histopathology. Figure 5 shows the H&E-stained re-cuts of the three tumor samples – primary, first recurrence, and second recurrence. Though morphologies differ considerably from primary to first recurrence to second recurrence, each individual image shows a fairly uniform pathology. Thus, the histopathology is somewhat indicative of the underlying molecular landscape, but is not sufficiently sensitive to recapitulate all of the mutational heterogeneity.

### A model for gliomagenesis and recurrence in this patient

The spatial and temporal heterogeneity characterized in our study (Figure 4) facilitates the construction of a putative model specific to this patient's individual disease. Prior to treatment, a subclonal population arose with a *PTEN* mutation, present in approximately half of the tumor cells. This mutation was probably at least partially driving the primary tumor, given the multiple previous reports of the same codon being mutated in GBM as well as the substitution's deleterious classification (see above). The primary tumor also harbored a *PIK3CA* mutation in a small subset of cells. No published studies report this codon as being mutated, however, and the classification algorithms suggest that it is likely a passenger mutation. In any case, the *PTEN* and *PIK3CA* mutations appear in separate subclonal populations, as evidenced by their mutual exclusivity in the first recurrence (with focus A harboring the *PIK3CA* lesion and focus B the *PTEN* lesion). Although the initial round of surgery, chemotherapy, and radiation did not eradicate the cells with the driver mutation, the second round appears to have done so. Indeed, the second recurrence has no evidence of *PTEN* mutation, but the subclonal population harboring the *PIK3CA* mutation seems to have acquired a “hypermutator" phenotype, acquiring three new observed (and certainly many more unobserved) mutations. It should be noted that we observed no mutations in recurrence A among the interrogated genes, underscoring the potential pitfall of drawing broad conclusions from only a specific region of a heterogeneous tumor.

## Discussion

Our work here highlights heterogeneity within tumors and their cellular subpopulations, and also describes mutational differences between primary tumors and recurrences. Although sets of primary tumors and metastases have been previously studied by some groups for genomic heterogeneity [1], [2], [27], analogous studies of sequential recurrences as presented here are uncommon. This is due to the difficulty of finding matched sets of properly preserved samples and the necessity of very high depth sequencing coverage across all samples in order to detect mutations that appear only in a minority of cells in a sample. The signals from such mutations are often below the noise threshold in Sanger sequence reads [21], and therefore likely remain underreported in the literature. We were able to accurately call mutations that were present in frequencies as low as 10%. Interestingly, the 10% threshold of detection was also cited in a recent study [28] of heteroplasmy in mitochondrial genomes using next-generation sequencing.

The results presented here establish several important principles. First, it is crucial that researchers do not rely solely on capillary-based sequencing for mutation detection and validation, since molecular heterogeneity may obscure important lesions. Second, filtering out annotated SNPs from lists of putative somatic substitutions can lead to false negatives because the mutation may occur at a known SNP. Some sites are assigned SNP IDs (i.e. rs numbers) based on scant validation, as is the case with the R282W substitutions reported here in *TP53*. The SNP (rs28934574) was reported only by one group in a single sample in dbSNP (www.ncbi.nlm.nih.gov/projects/SNP/). Even well-validated SNPs may be the site of somatic mutation simply by chance. Third, a single sample of tumor tissue might not be representative of other regions within the tumor (and may itself be an amalgamation of subclonal populations), and primary tumor samples do not necessarily represent the clonal origin of future recurrences.

Our proof-of-principle study required only one lane of sequencing on the Illumina Genome Analyzer II (at a current cost of approximately $1,000) because we focused on well-known targets of mutation in GBM. However, the method would be straightforward to scale up with regard to patient sample size and number of genes tested, particularly as costs decline and selective DNA capture methods develop. Additionally, the cellular resolution achieved here will improve – recent publications have demonstrated the promise of the ultimate in cellular resolution by sequencing at the single-cell level [3]. With these and other near-term technological advancements, extensions of the approach we have presented here will allow further progress toward the goal of personalized medicine via individual tumor sequencing.

## Supporting Information

Figure S1 Sequence coverage by gene and sample. The top panel shows coverage at each gene broken down by sample. The bottom panel shows the same quantities scaled to have the same average per gene, for visibility.
(DOC)

Figure S2 Sequencing of individual clones to validate mutations (site indicated with arrow) revealed by next-generation sequencing.
(DOC)

Table S1 Primer pairs used to amplify exonic regions.
(XLS)

Table S2 Candidate single nucleotide variants revealed by next-generation sequencing.
(XLS)
