# Supplementary figures and images for: Characterizing Mutational Heterogeneity in a Glioblastoma Patient with Double Recurrence
Source: PLoS One. 2012 Apr 20;7(4):e35262. doi: 10.1371/journal.pone.0035262 (PMC3335059; doi:10.1371/journal.pone.0035262)

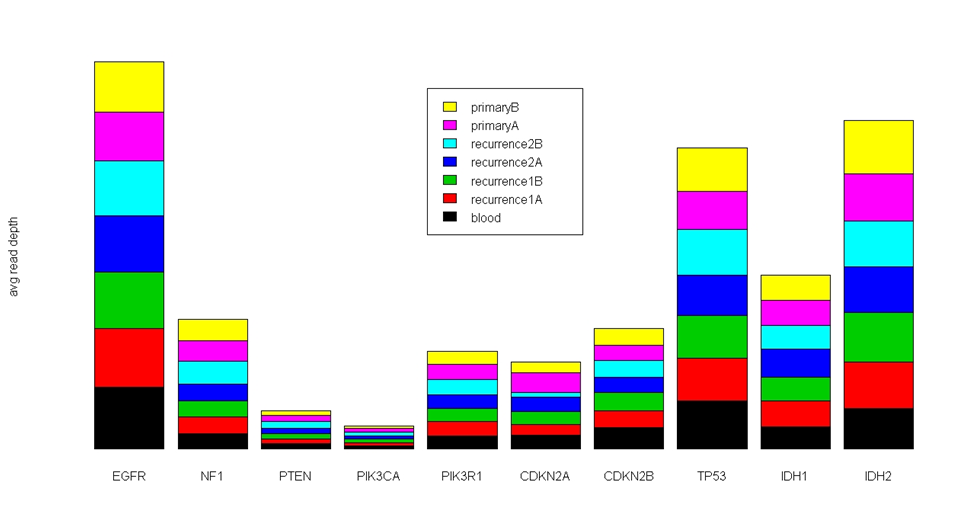

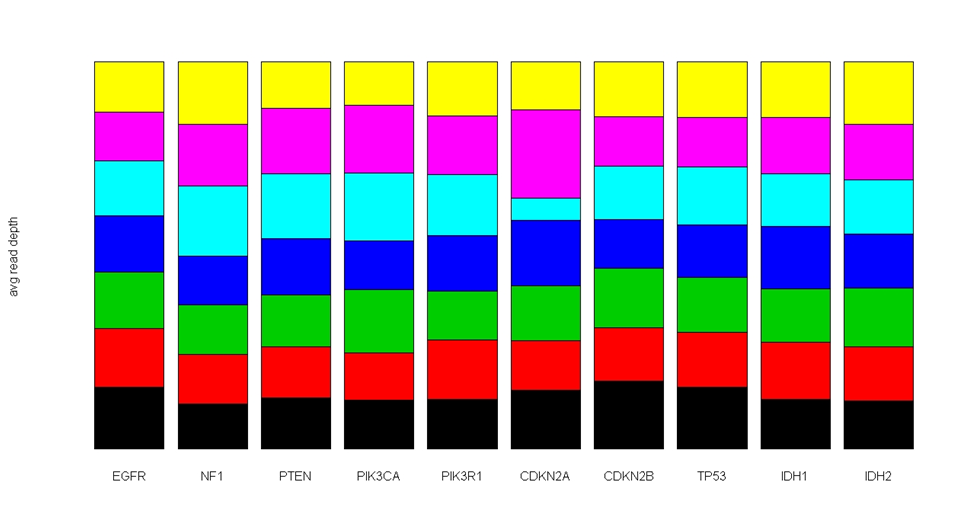

Supplement: Figure S1 — Sequence coverage by gene and sample. The top panel shows coverage at each gene broken down by sample. The bottom panel shows the same quantities scaled to have the same average per gene, for visibility. (DOC) [file pone.0035262.s001.doc]
