# Supplementary material for: Characterizing Mutational Heterogeneity in a Glioblastoma Patient with Double Recurrence
Source: PLoS One. 2012 Apr 20;7(4):e35262. doi: 10.1371/journal.pone.0035262 (PMC3335059; doi:10.1371/journal.pone.0035262)
Supplement: Figure S2 — Sequencing of individual clones to validate mutations (site indicated with arrow) revealed by next-generation sequencing. (DOC) [file pone.0035262.s002.doc]

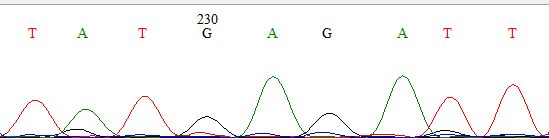


PTEN chr 10, bp 89643788, first recurrence B: Clone with mutation


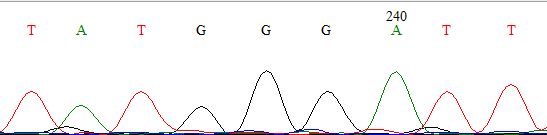


PTEN chr 10, bp 89643788, first recurrence B: Clone without mutation


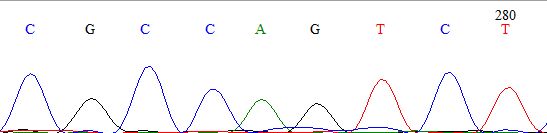


TP53 chr 17, bp 7517819, second recurrence B: Clone with mutation


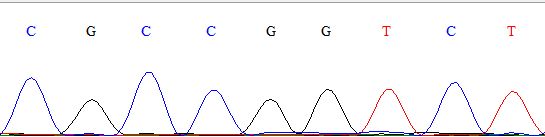


TP53 chr 17, bp 7517819, second recurrence B: Clone without mutation


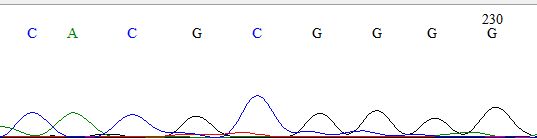


TP53 chr 17, bp 7520197, second recurrence B: Clone with mutation


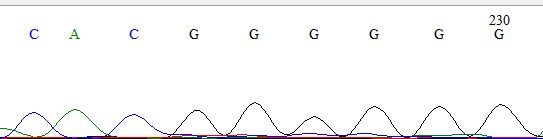


TP53 chr 17, bp 7520197, second recurrence B: Clone without mutation
